# Pandemic Shadows: Unmasking Gender Disparities in Academic Productivity and Work‐Family Conflict

**DOI:** 10.1111/cars.70038

**Published:** 2026-05-13

**Authors:** Marisa Young, Nicole McNair, Gabriella Christopher, Loa Gordon

**Affiliations:** ^1^ McMaster University Hamilton Canada; ^2^ Dalla Lana School of Public Health University of Toronto Toronto Canada

## Abstract

Women academics experience inequalities across multiple facets in the university context, including research productivity; teaching, advising and mentoring responsibilities; service workload; and cross‐over stress between work and family obligations. The COVID‐19 pandemic exacerbated these gender disparities. Drawing upon 2021 data from 467 research faculty and staff at a lead research university in Canada, we highlight such inequalities. While descriptive statistics suggest that men and women report similar perceived setbacks in productivity due to COVID‐19, results from structural equation models (SEM) estimating mediating associations tell a different story. These analyses demonstrate that women were disadvantaged compared to their male counterparts during the pandemic because of unequal exposure to work‐family conflict, which strongly correlates with the perception of lost productivity. We further hypothesized that faculty and research staff with young children would perceive lowered productivity during the pandemic, and that these burdens would be stronger for women than men. However, we did not find evidence to support these predictions. We unpack these results and conclude our paper with a discussion about the importance of rethinking the operationalization of “productivity” in post‐secondary institutions, given women's differential exposure and vulnerability to stressors during the pandemic.

## RÉSUMÉ

Les femmes universitaires sont confrontées à des inégalités sur plusieurs fronts dans le milieu universitaire, notamment en matière de productivité de la recherche, de responsabilités d'enseignement, d'orientation et de mentorat, de charge de travail liée aux services, ainsi que de stress lié à la conciliation entre obligations professionnelles et familiales. La pandémie de COVID‐19 a exacerbé ces disparités entre les sexes. En nous appuyant sur les données de 2021 recueillies auprès de 467 membres du corps professoral et du personnel de recherche d'une grande université de recherche canadienne, nous mettons en lumière ces inégalités. Alors que les statistiques descriptives suggèrent que les hommes et les femmes font état de baisses de productivité perçues similaires dues à la COVID‐19, les résultats des modèles d'équations structurelles (SEM) estimant les associations de médiation révèlent une réalité différente. Ces analyses démontrent que les femmes ont été désavantagées par rapport à leurs homologues masculins pendant la pandémie en raison d'une exposition inégale au conflit travail‐famille, qui est fortement corrélée à la perception d'une perte de productivité. Nous avons en outre émis l'hypothèse que les enseignants et le personnel de recherche ayant de jeunes enfants percevraient une baisse de productivité pendant la pandémie, et que ces contraintes seraient plus lourdes pour les femmes que pour les hommes. Cependant, nous n'avons pas trouvé de preuves à l'appui de ces prédictions. Nous décortiquons ces résultats et concluons notre article par une discussion sur l'importance de repenser l'opérationnalisation de la « productivité » dans les établissements d'enseignement supérieur, compte tenu de l'exposition et de la vulnérabilité différentes des femmes face aux facteurs de stress pendant la pandémie.

## 1 Introduction

In the spring of 2020, as the COVID‐19 pandemic spread throughout North America, public health measures required universities across Canada to close their doors (Kerr et al. 2022; Smith 2021). As post‐secondary educational institutions transitioned to an online learning and work environment, the sudden changes precipitated by the pandemic across the country had immediate impacts on all members of academia (Malisch et al. 2020; Shalaby et al. 2021). University faculty and research staff experienced—and continue to experience—unanticipated and costly impacts due to facility closures; policy changes and mandates; lack of access to research participants; and a shift to a predominantly online work‐world (Deryugina et al. 2021; Oleschuk 2020; Staniscuaski et al. 2021).

The current paper speaks to these unprecedented workplace circumstances and unforeseen stressors that inundated faculty and research staff across post‐secondary institutions. We directly build on the call for research on the impact of COVID‐19 on university faculty (Oleschuk 2020). In her 2020 *Canadian Review of Sociology* article, Oleschuk discusses the consequences of COVID‐19 on faculty productivity in the initial months of the pandemic. This highly cited opinion piece brought attention to the gendered impacts women faculty and research staff would experience in the face of the COVID‐19 pandemic, due to pre‐existing systemic barriers in the academy combined with the additional work‐family stressors, which, on average, disproportionately affect women. Our study helps answer Oleschuk's call by addressing two important research questions: (1) How have faculty and research staff been affected by COVID‐19 in terms of their research productivity and work‐family conflict? And, (2) How do these circumstances differ for women and parents of young children?

The current study draws upon *The Impact of COVID‐19 on University Faculty and Research Staff Survey*, which was disseminated at a large research‐focused university in Canada, to better understand how faculty and research staff have been affected in terms of research productivity and work‐family conflict. We operationalize research faculty and staff “productivity” by self‐reports of perceived ability to publish scholarly outputs and continue working on research despite COVID‐19. Work‐family conflict is defined as the extent to which one's work obligations interfere with their family or unpaid domestic obligations (Bond et al. 2003; Gordon et al. 2024.).^1^^,^ 2 Experiences of work‐family conflict, its antecedents, and outcomes have often been examined through simple comparisons of mean scores or Ordinary Least Squares regression analyses (Fettro and Nomaguchi 2018; Pedersen and Kilzer 2014; Schieman et al. 2009; Scheibling et al. 2025). However, such approaches overlook the fact that both these factors are multidimensional constructs influenced by multiple, interrelated factors. To capture this complexity and move beyond surface‐level item comparisons, we adopt a structural equation modelling (SEM) framework that allows us to examine both perceptions of productivity and work‐family conflict as latent constructs and formally test the processes through which gendered disparities emerge.

Findings from traditional mean‐difference tests suggest that women and men faculty reported similar perceptions of reduced research productivity during the COVID‐19 pandemic. Yet, results from our SEM show that women were, in fact, worse off: women experienced greater work‐family conflict than men compared to before the pandemic, and this stressor fully mediated the relationship between gender and perceived productivity losses. In other words, our findings suggest that women's pandemic‐era productivity setbacks were driven by unequal exposure to work‐family conflict. Having young children at home did not significantly change our focal associations, nor did these associations differ significantly by gender, contrary to expectations. We discuss the importance of these findings and a potential revisioning of “productivity” to ensure that academic women are not further impacted, given the gender‐specific setbacks they experienced during COVID‐19.

## 2 Literature Review

### 2.1 Academia, Productivity & Gender

Prior to COVID‐19—and still now—women in academia are faced with gender‐based inequities, leading to detrimental impacts on their research productivity, tenure and promotion, and recognition by status among universities’ higher‐ranking officials. Ample evidence highlights those various systemic expectations—both implicit and explicit—place unequal burdens on women in all facets of academia, including but not limited to: research productivity and output; teaching, advising and mentoring responsibilities; service workload; and emotional labour (Gordon et al. 2024; Malisch et al. 2020; Shalaby et al. 2021; Staniscuaski et al. 2021).

These trends date back over two decades, when Budig and England (2001) first published on the *motherhood wage penalty*. In academia, this factually based penalty is exemplified where some gaps in women's research and productivity may be linked to an unequal distribution of family and childcare responsibilities, at times resulting in decreased financial compensation (see Budig and England 2001; Deryugina et al. 2021). With increased care responsibilities at home, academic mothers are socialized to believe they must “choose” (within constrained options) between their work and their family obligations (see work by Deryugina et al. 2021; Staniscuaski et al. 2021; Yildirim and Eslen‐Ziya 2020). Amongst research faculty and staff, these supposed choices support the notion that career‐advancing academic productivity—measured by quality and quantity of academic publications and research funding income—is largely incompatible with child rearing (see Minello 2020, cited in Yildirim and Eslen‐Ziya 2020).

However, consequences for women extend beyond the wage‐penalty in the academic context and are sometimes not even linked to having children. For example, even before the pandemic, research has established that women academics generally receive less funding approval and smaller grants; are less frequently primary authors; are less likely to sit on peer review boards; are less likely to be approved by peer‐reviewed journals; are more likely to have teaching responsibilities; are more likely to provide support to students and research collaborators; and are expected to be more actively involved in service‐based activities than men, further reducing time spent on their research and publications (Shalaby et al. 2021). These obstacles were ultimately exacerbated with the COVID‐19 pandemic, where women in academia faced additional barriers affecting their career trajectories and opportunities than male‐counterparts, resulting in impacts on their work productivity, as well as various social, family, economic, and psychological‐based consequences (Deryugina et al. 2021; Malisch et al. 2020; Shalaby et al. 2021; Staniscuaski et al. 2021).

Despite many academic institutions implementing equity‐based practices to address gendered barriers, during unprecedented times—like a pandemic—these equity initiatives become at risk of being deprioritized when changes occur quickly, and institutions default back to biased policies and decision‐making processes (see Yu 2016, cited in Malisch et al. 2020). We would argue that the abruptness of the COVID‐19 pandemic and the shift to an online, work‐from‐home environment not only deprioritized many of these equity initiatives as COVID‐19 policies and protocols took precedence, but also *potentially added* new challenges amplifying the burdens faced by women, especially in terms of the work‐home interface. These additional work‐family conflict stressors are likely negatively associated with productivity.

### 2.2 Work‐Family Conflict, Productivity, Gender & Parental Status

From the onset of the COVID‐19 pandemic, extensive research has focused on gendered inequities women have faced, due in large part to domestic labour and caregiving responsibilities (Graham et al. 2021; Gulotta et al. 2021; Smith 2021). This is far from a novel pattern in North America. Arlie Hochschild's decades‐old notion of the ‘second shift’ underscores how unpaid childcare and housework on the one hand and paid work in the labour market on the other are at quintessential odds for women (Hochschild and Machung 1989). This concept remains unsurprising, given that women in North America bear an additional domestic burden and are obliged to uphold familial responsibilities and domestic care (Blair‐Loy et al. 2015; Yildirim and Eslen‐Ziya 2020).

COVID‐19‐related research extended Hochschild's theoretical framework, coining the *triple burden* now faced by working women where they encounter demands to remain productive in the workplace, uphold home care, and additionally maintain community duties (Gulotta et al. 2021; Smith 2021). While less research has been done to support this extension of thought, it is likely that the triple burden might apply to women in research faculty‐related positions. For example, research over the past decade suggests that, while the gender gap is closing, women are still performing the majority of these tasks overall (Ball and Daly 2012; Milkie et al. 2025), even while working full‐time positions in elite occupations, like those in academia (Gulotta et al. 2021; Yildirim and Eslen‐Ziya 2020). Notably, in cases where fathers undertook additional domestic labour in the early waves of the pandemic as daycares and schools closed, fathers’ engagement in childcare duties rarely persisted in the longer term (Fuller et al. 2025).

In the initial stages of research examining the impact of COVID‐19 on the work‐family interface, widespread literature focused on the additional challenges faced by women, more generally. In fact, early research drawn from several countries and focusing on the experiences of women in general—regardless of occupation—reinforces COVID‐19's detrimental impacts for women. Most of these outcomes are a product of increased domestic and familial obligations, as well as caretaking for children, aging parents, and other family and community members (Dawes et al. 2021; Gulotta et al. 2021; Smith 2021; Yildirim and Eslen‐Ziya 2020). Although survey research in the early days of the pandemic focused on analyzing the division of labour between heterosexual parents in families with children to see the gendered impacts on work‐life balance, Oleschuk (2020) noted that research specifically focused on academics and their work‐family demands was not prioritized at the time.

Women in academia, however, were not exempt from these added burdens and face (or faced) similar challenges to their careers resulting from their unequally allocated domestic and unpaid care work (Gulotta et al. 2021; Shalaby et al. 2021; Staniscuaski et al. 2021; Yildirim and Eslen‐Ziya 2020). It is likely that these disruptions impacted women faculty members’ productivity over the period of the pandemic, as well. More recent literature and research focusing specifically on academics confirms that the COVID‐19 pandemic placed additional burdens on these women and their familial and caretaking roles both in and out of the workplace (Deryugina et al. 2021; Shalaby et al. 2021; Yildirim and Eslen‐Ziya 2020).

Since the onset of the pandemic, women academics have spent, on average, fewer hours than their male counterparts on research time because of these additional burdens (Deryugina et al. 2021). For example, in their original survey of academics from multiple countries, Yildirim and Eslen‐Ziya (2020) provided results that yielded an alarming gap in the ways COVID‐19 has affected the working conditions of women academics with children. Yildirim and Eslen‐Ziya (2020) highlighted that being a parent during the pandemic led to difficulties for both women and men. For academics, however, they noted that productivity only diminished for women with children, not men. This association is likely a product of the work‐family conflict associated with having children and the unequal caregiving burdens placed on women over the course of the pandemic.

Women academics with younger children may have faced especially acute work‐family conflict during the pandemic because young children typically require more intensive supervision, hands‐on caregiving, and assistance with daily routines than older children (Wikle and Cullen 2023). During school and childcare closures, these demands likely expanded further as parents of young children had to absorb responsibilities ordinarily shared with schools, childcare providers, and other institutional supports, while still managing to fulfill their ongoing work responsibilities (Dawes et al. 2021; Wade et al. 2021; Yildirim and Eslen‐Ziya 2020). More specifically, the COVID‐19 pandemic created unique challenges for young children in preschool, kindergarten and early primary grades, where learning typically occurs through teacher‐assisted, activity‐based programming (Hu et al. 2021; Spadafora et al. 2023; Timmons et al. 2021). With the shift to online learning, young children required assistance navigating technology and software programs to access their learning materials, while also needing instructional delivery to understand their learning goals and expectations (Nikolopoulou 2022; Spadafora et al. 2023). Additionally, with inconsistent mandates for online instruction time, a lack of technological preparedness by early learner instructors, and shorter attention spans of young learners, online lessons during COVID‐19 school closures typically resulted in fewer weekly hours of structured learning for young children than their in‐person programming prior to the pandemic (Hu et al. 2021; Spadafora et al. 2023; Timmons et al. 2021).

By contrast, older children are often more independent in their daily routines and schooling. Therefore, even if online instructional time was reduced during the pandemic for older students, they could be assigned more complex and time‐consuming tasks, projects and homework that could also be completed independently (Dawes et al. 2021). These factors may reduce the immediacy and intensity of parental oversight for older children. At the same time, not all research finds the expected differences. A recent study by Schieman et al. (2021) found that parental gender did not determine differential reports of work‐family conflict. Moreover, parents of young children (0–12 years) reported similar conflict compared to pre‐pandemic periods, while those with older children (13 to 18 years) reported lower levels. We take these contrary findings into consideration and compare experiences by gender and across parents with children of varying ages.

Taken together, previous literature leads us to expect that faculty and research staff's work‐family conflict and productivity were shaped by the COVID‐19 pandemic. We cannot, however, formally test changes from pre‐pandemic levels with the present data. We instead pose the following testable hypotheses given our analytic design.

Hypothesis 1 Faculty and research staff's perceived research productivity has been negatively affected by the work‐family conflicts brought on by the COVID‐19 pandemic.

Hypothesis 2a Faculty and research staff with younger children in the household will report greater work‐family conflict and lower perceived productivity than those without younger children.

Hypothesis 2b The negative associations linked to having young children will be stronger for women than for men.

## 3 Data & Methodology

### 3.1 Survey Data Collection & Sample Characteristics

The data are from *The Impact of COVID‐19 on University Faculty and Research Staff Survey*, which was disseminated at a large research‐focused university in Canada, aimed at better understanding how faculty and research staff have been affected in terms of research productivity, work‐family conflict, and overall well‐being. The survey was limited to those who identified as faculty members or research staff. Research staff were defined as post‐doctoral fellows, research fellows, or research associates (∼25%). The population of faculty and research staff were identified by the organizations’ research and analysis institute, which has the contact information for all faculty and research staff identified by those categories.

The survey of faculty and research staff was administered at two time points. Timepoint 1 was launched on June 23rd, 2021, with a reminder email sent on June 29th, and the survey closed on July 4th. Upon closing, 20% of the non‐responders were randomly selected for a Hard‐to‐Reach follow‐up phone call. 50% of these potential respondents received phone calls on July 5th, and the other 50% received phone calls on July 6th. The Hard‐to‐Reach survey closed on July 11th. Timepoint 2 was launched on June 30th, with a reminder email sent on July 6th, and the survey closed on July 11th, 2021. We followed the same procedure as the first wave: upon closing, 20% of the non‐responders were randomly selected for a Hard‐to‐Reach follow‐up phone call. 50% of these potential respondents received phone calls on July 12th, and the other 50% received phone calls on July 13th. The Hard‐to‐Reach survey closed on July 18th. This research was approved by the University's Research Ethics Board (#5483).

We received a response rate to the survey of 34% (*n* = 475; 467 respondents were included in final analyses after data cleaning), with half identifying as men and half as women. The majority of respondents were in the Faculty of Health Sciences (26%) followed by Science (22%), Engineering (18%), and Social Sciences (15%). 38% had been employed at the University for five years or less. 42% reported being at the University for more than six years. This aligns with the average age of the sample, which was between 41 and 51 years old. Assistant professors made up 16% of the sample, Associate professors 21%, and Full professors 30% of the sample. We did not, however, stratify or test differences between tenured and non‐tenured faculty. Nor did we look at comparisons between faculty and research staff. Our decisions to not test these potentially relevant intersecting circumstances were based on sample size and power analyses, given that these limitations directly compromise the sensitivity of SEM analyses.

### 3.2 Focal Survey Measures & Descriptive Results

#### 3.2.1 Perceived Research Productivity

We asked respondents several questions about their current productivity compared with prior to the pandemic. These questions addressed a series of topics, including the impact of COVID‐19 on respondents’ perceived ability to publish scholarly outputs and continue working on research despite COVID‐19. We used three items to capture these perceptions: (a) published outputs; (b) publishing opportunities; and (c) continued scholarship in the face of COVID‐19.

To measure *published outputs*, we asked whether respondents’ published scholarly outputs had been “much less” (1), “less” (2) “same” (3), “more” (4), “much more” (5) compared to before the onset of the COVID‐19 pandemic.

We measured *publishing opportunities* by asking respondents whether they “have missed opportunities to publish scholarly outputs due to increased family obligations and/or health concerns as a direct result of COVID‐19 and its impacts.” Response categories included “much less” (1), “less” (2) “same” (3), “more” (4), “much more” (5). We reverse coded this measure so that higher scores reflected more opportunities to publish.

Finally, we measured *continued scholarship in the face of COVID‐19* by asking respondents to indicate the degree to which they “had to stop working on an existing scholarly output” compared to before the onset of the pandemic. Response categories included “much less” (1), “less” (2) “same” (3), “more” (4), “much more” (5). We reverse coded this measure so that higher scores reflected more continued work in the face of COVID‐19.

#### 3.2.2 Work‐Family Conflict

We asked respondents about their current perceived experiences of work‐family conflict compared with prior to the pandemic. See Note 1, where we outline justification for measuring work‐to‐family conflict instead of family‐to‐work conflict. We adapt four well‐known survey items used to measure work‐family conflict (see Bond et al. 2003; Gordon et al. 2024; Schieman et al. 2009). Each question was prefaced with: “In comparison to what it was like prior to March 2020 – before the onset of the pandemic (a) How often have you not had enough time for your family or other important people in your life because of your job?; (b) How often have you not had the energy to do things with your family or other important people in your life because of your job?; (c) How often has your job kept you from doing as good a job at home as you could?; and (d) How often has your job kept you from concentrating on important things in your family and personal life?” Responses comprised, “much less” (1); “less” (2); “same” (3); “more” (4); or, “much more” (5).

#### 3.2.3 Central Covariates

Our second set of hypotheses predicts that respondents with young children have experienced the pandemic differently when it comes to productivity and work‐family conflict, and that these patterns may be more pronounced for women than men. We code gender as (1) women, compared to men (0, comparison category).

We created a measure of children in the household that captures whether respondents have (a) at least one child under 6 in the household; (b) at least one child 6 to 12 in the household, and (c) at least one child 13 to 18 in the household. Respondents who scored ‘0’ on all of these measures have no children under 18 in the household.

#### 3.2.4 Additional Covariates

We controlled for a variety of covariates in all analyses, including: *Years in current position*. We asked respondents, “How many years total have you been employed by the University (including parental, medical, and other leaves)?” Response categories included: Less than 6; 6–10; 11–15; 16–20; 21–25; 26–30; greater than or equal to 30 years.

*Marital status*. We compare those who are married or common law (1) to all other relationship status categories (0, comparison category). This measure was derived from the household composition questions: those who indicated they were living with at least one other person and identified that person as a spouse/partner received a “1” on this measure.

## 4 Data Analyses

### 4.1 Descriptive Statistical Analysis

We first examined whether traditional approaches to evaluating gender differences in productivity—such as comparing item‐level means using *t*‐tests—would reproduce disparities reported in prior research. Table 1 presents descriptive statistics for each productivity item by gender to illustrate what these standard comparisons typically reveal. While widely used, this approach is limited because it relies on observed items that contain measurement error and does not account for the underlying latent structure of productivity or its association with work‐family conflict. As a result, these mean differences can mask or distort the patterns we aim to understand, particularly in the context of the COVID‐19 pandemic. Our SEM framework addresses these limitations by modelling productivity as a latent construct and explicitly incorporating key mediating processes, allowing us to interrogate gender differences in a more rigorous and conceptually coherent manner.

**TABLE 1:** Descriptive statistics for productivity variables & standard mean‐difference tests.

	Women		Men	
Variable	Mean	SD	Mean	SD
**Productivity**				
Published outputs	2.432	0.901	2.481	0.979
Publishing opportunities	2.186	1.009	2.494^**^	1.082
Continued scholarship	2.265	0.973	2.465	0.966

*Note*: *p < 0.05, two‐tail *t*‐test or chi‐square for binary outcome.

### 4.2 Structural Equation Models

To further probe the relationships between gender, work‐family conflict, and productivity, we employed SEM. This analytic approach offers two key advantages: it allows us to model latent variables based on multiple observed indicators, reducing measurement error, and it enables estimation of both direct and indirect (mediated) pathways within a single framework (Kline 2016). In doing so, we move beyond regression‐based associations to examine the mechanisms linking social location to well‐being and work outcomes during the pandemic. Finally, SEM offers robust model fit statistics, ensuring that our hypothesized conceptual model reflects the empirical data structure, while controlling for measurement error and covariates like job tenure and marital status – as is the case in the current analysis. This integrated approach enhances the validity and interpretability of our findings in ways that simpler regression models cannot. We conducted all SEM analyses in Stata 18.5 with bias corrected bootstrapping using 10,000 samples (Cain 2021). We estimated analyses using full‐information maximum likelihood with missing values (MLMV), which allows all available data to contribute to the model without listwise deletion. This approach produces unbiased parameter estimates under missing‐at‐random assumptions and is considered the recommended method for handling missing data in SEM (*N* = 467).

**FIGURE 1:**
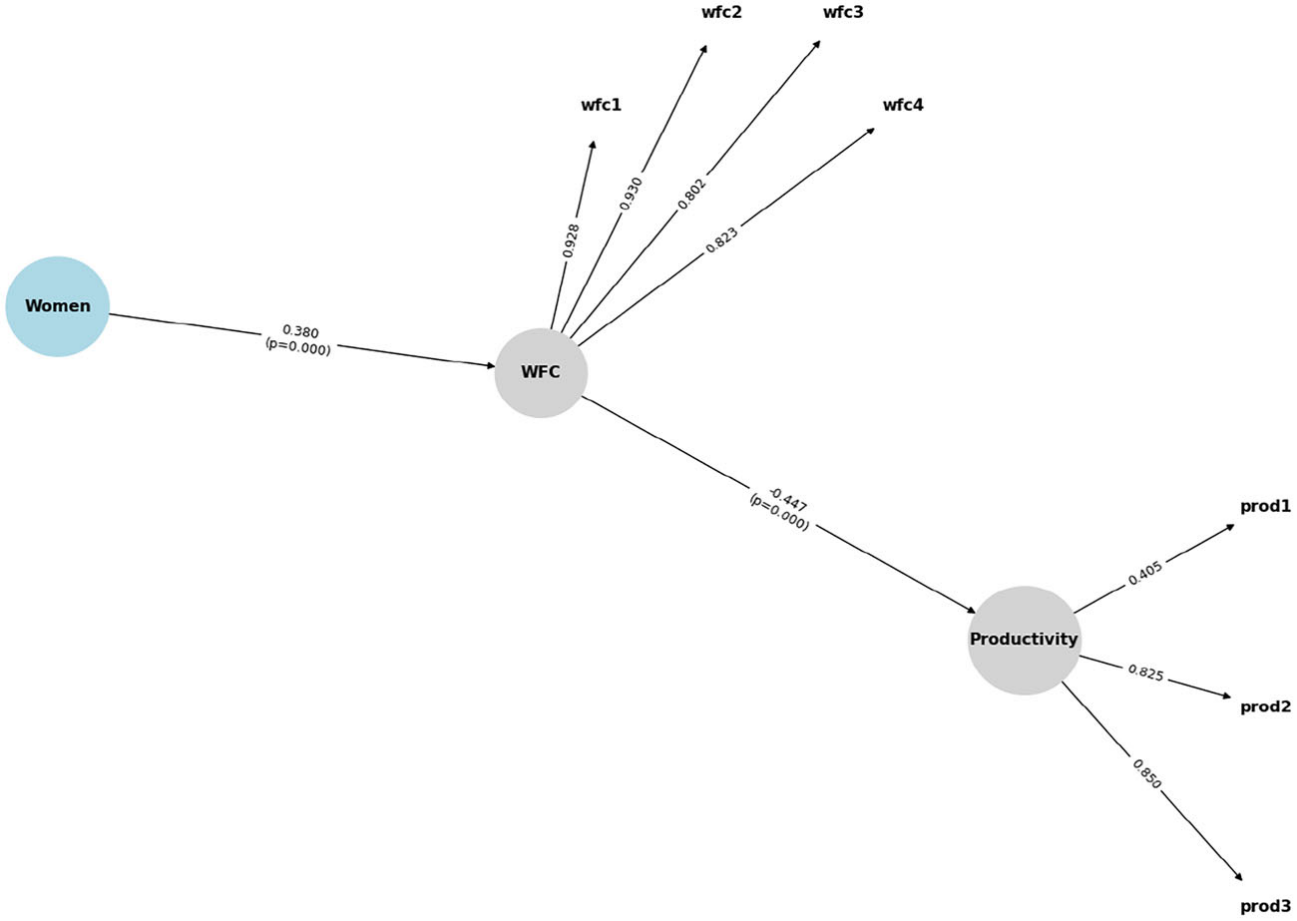
Structural equation model of direct and indirect effects of gender on work‐family conflict and productivity (significant variables, only) [Colour figure can be viewed at wileyonlinelibrary.com] Notes: WFC = Work‐family conflict. Standardized coefficients presented.

We estimated two SEM models. The first estimates the effect of gender on our latent measure of productivity. The second estimates the mediated association between gender, our latent measure of work‐family conflict, and productivity. We present results from the latter model in Figure 1. We also evaluated whether having younger children was associated with greater work‐family conflict and lower perceived productivity, and whether these associations differed by gender. We discuss these results further in the next section.

## 5 Results

In Table 1, we demonstrate that the mean differences estimated with standard *t*‐tests suggest that men and women (pooled across parental status) report similar perceived productivity, except for publishing opportunities. Men report greater perceived publishing opportunities (mean_women =_ 2.186, mean_men_ = 2.494, *p* < 0.01). Despite these gender similarities, it is worth noting that both genders reported an average score of less than ‘3’ (i.e., productivity stayed the same), which suggests that perceived productivity decreased overall because of the pandemic.

### 5.1 Structural Equation Model Results

We first estimate the effect of gender on our latent measure of productivity. This model is pre‐emptive and does not provide sufficient information about the association, given its simplistic approach. However, we do note that the main association between gender and productivity is negative and statistically significant (*b* = −0.086, *p* < 0.041), suggesting that women reported lower perceived productivity because of the COVID‐19 pandemic. This model, however, does not fit the data well (results available upon request), suggesting that there are more elements necessary to explain the variance in productivity responses.

Our second SEM model provides a better fit to the data. We observed a significant chi‐square value (χ^2^ = 125.92, *p* <  .001), which is not uncommon in bootstrapped SEM results, or with larger sample sizes. We also evaluated other fit statistics to provide additional confidence, including the comparative fit index (CFI), the Tucker‐Lewis index (TLI), and the Root mean square error of approximation (RMSEA). These values suggest conservative support but are within the range of acceptability for SEM fit (CFI = 0.915; TLI = 0.869; RMSEA = 0.087, 90 % CI = 0.070–0.105) (see Kline 2016; West et al. 2023).

Figure 1 presents direct and indirect effects for focal variables only (standardized coefficients presented). All results present bias‐corrected bootstrapped significant tests. Table 2 provides both standardized and unstandardized coefficients for all analytical variables, separated by direct, indirect, and total effects.

**TABLE 2:** Tests of the indirect, direct, and total effects of the focal independent variables predicting productivity in the path model.

Focal Independent Variables			
Women	Unstandardized coefficient	Standardized coefficient	% of total effect
Indirect effect through WFC	−0.130^**^	−0.170	79.3
Direct effect	−0.034 (n.s.)	0.045	17.6
**Total effect**	−**0.164** ^*^	−**0.170**	**96.9**

*Note*: ^*^ *p* < 0.05; ^**^ *p* < 0.01; ^***^ *p* < 0.001 *(two‐tailed tests)*. Unstandardized (b) and standardized (B) coefficients are presented based on bootstrapped results (samples = 1000). Percent (%) of total significant effect excludes the negligent direct effect of gender on productivity. WFC = work‐family conflict. The model included the following considerations: Presence of kids under 6, presence of kids 6 to 12, presence of kids 13 to 18, marital status, and job tenure. Note, we tested interaction terms between the presence of young children and gender but did not find any evidence that one status depended on the other in predicting productivity directly or indirectly. The addition of the interaction effect reduced the model fit considerably and was excluded from the final analyses and results table.

Results from our SEM analyses indicate that, overall, most of the variance in women's lower perceived productivity is transmitted through the work‐family conflict pathway. This finding supports our first hypothesis. Being a woman predicts significantly higher work‐family conflict (*B* = 0.380, *p* < 0.001). Further, this pattern significantly affects productivity indirectly (indirect effect, *B* = −0.170, *p* < 0.01). In fact, approximately 80% of the mediated effect between gender and productivity operates through work‐family conflict. The direct “women → productivity” path is negligible (*B* = 0.045, n.s.). Thus, the total gender effect on productivity (*B* = −0.170) is almost fully mediated.

We next examined whether having a child under six years old was associated with productivity and work‐family conflict overall. Contrary to Hypothesis 2a, the presence of a young child had no significant direct effect on productivity, and we did not find support for the expectation that respondents with young children reported higher work‐family conflict. These results were somewhat surprising, but not entirely dissimilar to Schieman et al. (2021), who found that parents of young children reported similar levels of conflict compared to pre‐pandemic periods.

We then examined whether the implications of having a young child differed by gender. Contrary to Hypothesis 2b, our tested interaction terms between the presence of young children and gender did not show evidence that one status depended on the other in predicting productivity directly or indirectly, nor did these statuses combine to differentially shape levels of work‐family conflict. The addition of the interaction effect reduced model fit considerably and was therefore excluded from the final model. Again, we underscore the small sample size of our data, and the power limitations associated with those low cell counts—a note we address further in the limitations section. See Appendix B for a breakdown of counts by gender and presence of young children in the household.

Overall, the SEM reveals a clear stress‐mediation sequence—woman → higher WFC → lower productivity—that may account for the gender gap in pandemic‐era scholarly output. Hypothesis 1 is supported. Hypotheses 2a and 2b are not supported. Appendix A provides a breakdown of the SEM results with all measures (standardized coefficients presented).

## 6 Discussion

The COVID‐19 pandemic upended the university context in unimaginable ways. As these communities moved to online learning and working from home, there have been questions about the longer‐term consequences for faculty and research staff's work‐family conflict and subsequent productivity. The broader question addressed in our study points to the discordance between the rigid expectations of tenure and promotion, accolades, status achievement, and recognition in a context inundated with ambient stressors amplified by and associated with the pandemic.

We well know that some social and demographic groups experienced greater hardships when it came to work‐family conflict and decreased productivity, compared to others. Our study highlights only two of these groups: women—particularly mothers. In the most highly cited *Canadian Review of Sociology* article from the early days of the pandemic, Merin Oleschuk (2020) rightly calls for action to address the potential inequalities COVID‐19 has generated for women in particular, and mothers with young children secondarily. We answer this call by addressing two focal queries: (1) How have faculty and research staff been affected by COVID‐19 in terms of their research productivity and work‐family conflict? (2) How do these circumstances differ for women—particularly mothers with young children? We expected that the pandemic would impact productivity directly, but also indirectly by increasing work‐family conflict experiences. We further presumed these associations would be stronger for women, especially those with young children, compared to their male counterparts, and those without young children.

### 6.1 Gendered Results in Productivity and the (In)Significant Impact of Children

Standard mean‐difference tests showed that men and women reported similar impacts of COVID‐19 on their perceived productivity over the previous year, except for publishing opportunities, where men reported more. These findings were surprising, given prior research outlining that women likely had less time for conferences and grant writing. For example, women faculty generally devote more time to service work and are typically approached more frequently by students for mental health support than their male counterparts. In a time of uncertainty and with a transition to online learning, women faculty experienced increases in service work, teaching time, and providing student support (Malisch et al. 2020; Shalaby et al. 2021), resulting in less time to work on scholarly outputs. Additionally, women are underrepresented in academia (Deryugina et al. 2021), but particularly in STEM‐related fields (Shalaby et al. 2021), missing opportunities to participate in the influx of COVID‐19‐related research in the early days of the pandemic (Deryugina et al. 2021). We argued that the contrary findings from mean‐difference tests in our data compared to prior scholarship might therefore be an artifact of the statistical method. Our subsequent SEM analyses help unpack these unexpected findings: while standard approaches initially suggest that men and women encountered similar setbacks in productivity, women experienced far greater work‐family conflict due to COVID–19 ‐ a stressor that, in the context of the pandemic, is strongly associated with lost productivity during this period. In other words, even though it appears women and men have similar productivity, women were disadvantaged compared to their male counterparts during the pandemic because of unequal exposure to work‐family conflict, which ultimately correlates with lost productivity. These findings might not be clear if relying only on traditional mean‐difference tests or ordinary least squares approaches.

However, even within our SEM analyses, we do not find that respondents with young children—including both women and men—were directly disadvantaged in terms of reported productivity or work‐family conflict throughout this time compared to those without young children. This finding was contrary to expectation yet not unique in post‐COVID research. For example, Schieman et al. (2021) found that parents with children aged 0–12 reported similar conflict levels relative to pre‐pandemic periods. Those with older children, however, reported even less conflict than before this time. The authors also did not find differences dependent on gender and parental status. Our results align more closely with these authors’ data than with prior studies showing significant differences in conflict between mothers with young children and their respective counterparts.

This might be a product of several phenomena. First, we know that those who face work‐family conflict due to having young children are likely to scale back on work *regardless of the pandemic*. That is, parents of young children who find it difficult to manage competing work and family obligations might not have been impacted by the pandemic to the extent we suspect, given that these modifications to work—in the form of scaling back on work hours, demands and service—might have begun prior to the onset of COVID‐19 and therefore not reflected in our data (see Young and Schieman 2018).

Second, this might be coupled with the artifact of our methodological operationalization, given the wording of our survey questions. For example, we asked respondents to indicate the degree to which they have experienced changes in a given work‐family conflict compared to prior to the pandemic (much more, more, same, less, much less). It could be that parents with young children were already experiencing high levels of conflict, compared to those with older or no children. Therefore, results may reflect the possibility that some parents were no worse off than before, which might speak to the inequalities inherent in the university system disadvantaging those forced to combine conflicting work and family obligations, especially in high performance positions like those faced by tenure‐track faculty.

Finally, it is possible that spending more time with young children during lockdowns offered parents unexpected buffers—spontaneous play, frequent physical affection, and reduced loneliness—that counterbalanced the added caregiving demands and, for some faculty, lessened the overall stress typically associated with parenting young children. In subsequent qualitative analyses of round table discussions, these sentiments were voiced and might help explain our unexpected quantitative findings.

These explanations might not speak to differences in productivity by parental status and age of children, however. For this, we draw instead on recent work citing that those who have competing expectations might be less likely to participate in research, like the survey we launched. Young and colleagues (2023) provide a compelling case for selection bias in research on the work‐family interface, which we suggest can be extrapolated to the current study. When studying the strain on mental health and absence of “time” that often accompany competing roles, it is unlikely that those most vulnerable to these processes will take the time to document those experiences in a survey. It is likely that these time constraints were exacerbated during the pandemic, further reinforcing this phenomenon. We therefore suggest that the discordant absence of association between having young children and research productivity in the context of COVID‐19 might be a methodological and statistical artifact that needs to be better addressed by future researchers. Despite the plausibility of these arguments, it might also be that after a full year into the pandemic (when data for the current study was collected), parents had made changes to their combined and conflicting work and family demands that came about with the ambient stress of the pandemic.

### 6.2 Limitations of Parsimony

A key limitation of our study is its intentionally parsimonious measurement strategy. In SEM, including fewer observed variables is common practice when the sample size is moderate (*N* = 467) and the theoretical model already contains several latent constructs. When we experimented with adding theoretically plausible controls—most notably academic discipline and faculty rank—the overall fit deteriorated (results available upon request), suggesting the extra paths introduced more noise than explanatory power. Over‐parameterized SEMs also risk multicollinearity, unstable estimates, and capitalizing on chance, reducing generalizability (Kenny and McCoach 2003). Because of these concerns, we retained only the covariates most directly linked to our predicted stress‐process hypotheses (gender, presence of children across age categories, marital status, job tenure). While this parsimony safeguards model stability and interpretability, it leaves open the possibility that unmodelled role‐specific pressures—such as disciplinary lab closures or rank‐related service loads—could account for additional variance in pandemic‐era productivity. It also leaves open the possibility that unmeasured socioeconomic differences shaped both exposure to work‐family conflict and perceived productivity during the pandemic. Future research with larger, multi‐institutional samples should test extended models with these contextual factors without sacrificing fit quality.

### 6.3 Additional Limitations

We also note several additional limitations, mostly associated with our data. First, our study relies on cross‐sectional data, which limits our ability to draw conclusions about how work‐family conflict and productivity evolved over time during the pandemic. Although respondents reflected on changes relative to pre‐pandemic conditions, these retrospective assessments may be affected by recall bias. Longitudinal data would allow a more robust examination of these processes as they unfold.

A second limitation concerns statistical power. While the total sample size was adequate for the SEM approach, subgroup sizes—particularly for gender‐by‐parental‐status comparisons—were modest. These smaller cell sizes reduced the precision of estimates and limited our ability to detect interaction effects. Sample size limitations were previously noted in the results section with reference to Appendix B for a further breakdown of numbers by gender and presence of young children.

A third limitation concerns omitted confounding variables. We were unable to account directly for socioeconomic status or adjacent structural factors that may shape both work‐family conflict and productivity. While unmeasured confounders may impact the modeled associations, we anticipate these effects would be relatively small within the context of our population of university faculty and research staff; compared to the community at large, this population likely experiences less variability in socioeconomic factors (Jafari et al. 2024). However, as a result of this limitation, the associations reported here should not be read as a fully exhaustive causal account of gendered productivity differences during the pandemic.

Finally, the generalizability of our findings should be considered. The study draws on data from a single Canadian university. Institutional cultures, policies, and work environments vary widely across the post‐secondary sector. While we had a good response rate for online surveys (35%), our data may not represent the diverse experiences of the University's total faculty and research staff composition. As a result, the patterns we observe may not fully reflect the experiences of academic workers in other institutional contexts. Nevertheless, we would imagine that the faculty and research staff in our data reflect experiences of others in post‐secondary institutions across the country, and maybe even in the United States. Our analyses were prompted by a faculty member employed at an American institution who wrote a thoughtful piece drawing attention to the importance of productivity and work‐family challenges facing women in academia—Merin Oleschuk. Her impactful—highly cited—2020 commentary suggests that our findings may resonate with many across a variety of post‐secondary institutions.

### 6.4 Reimagining Productivity

Despite the noted limitations, our study underscores a series of take‐away questions and key messages. Considering the context that precipitated our study and Oleschuk's (2020) call for research on the impact of the pandemic on tenure, promotion, and productivity, we conclude with the question: What do more equitable approaches to productivity look like and how can we get there? As post‐secondary institutions have entered a period of post‐pandemic recovery, universities are (or should be) committed to supporting their faculty and research staff, in particular the communities who have been most burdened—only some of whom have been represented here. The survey that generated the data for this study was created as a solution‐oriented tool that would aid in the development of evidence‐based improvements for members of university research communities. We hope results and recommendations generated from this study can aid conversations across campuses in Canada and help implement strategies to facilitate (a) equitable opportunities and evaluation of productivity, and (b) better work‐family balance, especially for women most impacted by times of crisis like the pandemic.

## 7 Conclusion

Our research team initially set out to determine how women and parents with young children fared throughout the pandemic. What came of that endeavour is an important research study for post‐secondary administrators to embrace moving forward in a post‐COVID‐19 context. The impact of the pandemic on university faculty and research staff has been far‐reaching. While we have been unable to tackle all of the multifactorial nuances in the current study—related to race and ethnicity, nativity, age, sexuality, dis/ability, discipline, and tenure, for example—we do highlight one important take‐away message: women in academia have been impacted more than their male counterparts. While initial results based on standard mean‐difference tests suggest this is not the case when it comes to productivity, subsequent, more advanced analyses provide a clear picture that women's differential exposure to work‐family conflict inhibited their research productivity during and immediately after the pandemic.

Furthermore, we argue that the absence of differences between respondents with young children and those with older or no children aligns with more recent research on COVID‐19 and parents' work‐family conflict (Schieman et al. 2021). Our results might therefore not be as contrary as initially thought. However, the absence of difference among these groups does contradict a host of prior research outlining the differential burdens among women with small children (Dawes et al. 2021; Wade et al. 2021; Yildirim and Eslen‐Ziya 2020). We propose these findings might, in part, be an artifact of our methodological tool: these individuals may have either (a) scaled back on work hours and demands prior to the pandemic; (b) already experienced heightened work‐family conflict before the pandemic; (c) adapted to their conflicting work‐family experiences; or (d) opted not to respond to our survey. Of course, we have no possibility of testing these scenarios with the data available. Despite this, an important message translates through the current study: due to the pandemic, university faculty and research staff have experienced major productivity setbacks and elevated levels of work‐family conflict, and our data demonstrates that these patterns are worse for women than men. We hope the results from our study will prompt university leaders to act in support of their faculty and staff in a post‐COVID‐19 context, and in future periods of social upheaval and crisis.

## Funding

A grant award from the Social Science Humanities Research Council Canadian Research Chair Funds supports this study (Reference Number: CRC‐2019‐00301; Marisa Young, P.I).

## Endnotes

1 We operationalize role interference solely as *work‐to‐family conflict* rather than including the reciprocal *family‐to‐work* dimension. For research‐intensive faculty and research support staff, the flow of strain is overwhelmingly from work into the home sphere. Academic work is characterized by open‐ended tasks—grant preparation, manuscript revisions, student mentoring, and after‐hours e‐mail—that spill into evenings and weekends, blurring temporal boundaries between professional and personal life. The ethos of the “ideal academic” encourages constant availability and prioritization of scholarship, teaching, and service (Blair‐Loy 2005). As a result, employees frequently report sacrificing family time or personal recovery to meet career benchmarks, whereas the reverse—family obligations disrupting core academic responsibilities—occurs far less often and is typically self‐managed (Allen et al. 2014). We also considered including both in our SEM, but fit statistics suggested that family‐to‐work conflict be removed from the model.

2 The unobserved gender difference in having young children on work‐family conflict might be because these latter measures capture perceived change rather than actual pre‐pandemic levels. It may be that some groups, like those with young children, did not report much of an increase. This might explain why the parental status finding is weaker, if baseline scores were already high for parents.

## Appendix A

### A.1

Structural equation model of direct and indirect effects of gender on work‐family conflict and productivity (all model variables). Notes: WFC = Work‐family conflict. Standardized coefficients presented.

## Appendix B Crosstabulations by gender and presence of young children

### B.1

**Table:**

	Women		Men	
	Kids < 6	No Kids < 6	Kids < 6	No Kids < 6
Variable	*N* (%)	*N* (%)	*N* (%)	*N* (%)
Negative career trajectory	19(40.4)	98(52.7)	28 (59.6)	88(47.3)
Fewer publications output	12(35.3)	85(52.8)	22(64.7)	76(47.2)
Fewer grant submissions	8(38.1)	51(56)	13(61.9)	40(44)
Work‐family conflict *^a^*	17(53.1)	106(60.6)	15(46.9)	69(39.4)

*Note: ^a^* Binary measures used, where ‘1’ reflects higher levels of work‐family conflict, compared to lower levels.
